# Maternal History of Major Depression, Social Support from Peers, and Children’s Risk for Self-Injurious Thoughts and Behaviors

**DOI:** 10.1007/s10802-025-01363-5

**Published:** 2025-09-01

**Authors:** Sara I. Buseman, Brandon E. Gibb

**Affiliations:** Department of Psychology, Binghamton University, State University of New York, Binghamton, NY 13902-6000 USA

**Keywords:** Maternal depression, Social support, Self-injury, Suicidal ideation, Nonsuicidal self-injury

## Abstract

Self-injurious thoughts and behaviors (SITBs), encompassing both nonsuicidal self-injury and suicidality, are a growing public health concern in youth. Although maternal depression is a well-established risk factor for psychopathology in offspring, less is known about protective factors that may mitigate this risk. Peer social support, particularly during the transition to adolescence, may play a critical role in reducing risk for SITBs, yet limited research has examined the potential impact of different domains of peer influence (e.g., close friends versus classmates) as specific and distinct facets of the peer environment. In a two-year longitudinal study, we assessed SITBs in 215 children (ages 8–14), half of whom had mothers with a history of major depressive disorder (MDD) during their child’s life. We hypothesized that maternal MDD would predict increased risk of SITBs in children but that higher levels of peer social support would buffer this effect. Survival analyses confirmed that children of mothers with MDD were over twice as likely to develop SITBs during the follow-up. Importantly, higher levels of social support from classmates uniquely predicted reduced risk for SITBs in children, even after accounting for the influence of maternal MDD history. Our findings highlight the role of broad peer environments in protecting against SITBs in youth. These results underscore the importance of school-based interventions that foster social inclusion as potential preventive measures for SITBs.

Self-injurious thoughts and behaviors (SITBs) can be defined as a range of self-directed thoughts and actions performed intentionally to harm, including both nonsuicidal self-injury (NSSI) and suicidal self-injury (Nock, 2010). SITBs are an important public health concern, with rates of suicide and self-harm behaviors in pre-teens significantly increasing over the last 20 years (Castellví et al., 2017; Ruch et al., 2024). Recent estimates in non-clinical samples suggest that approximately 14% of children have a history of suicidal ideation (Lawrence et al., 2021) and 9% have a history of NSSI (Burke et al., 2023), with rates of SITBs increasing even further during adolescence (Hua et al., 2023; Esposito et al., 2023). However, despite the clear significance of SITBs in the late childhood to early adolescent period, relatively little is known about specific risk and protective factors across this developmental period. Research in youth is important given evidence that SITBs increase risk for an array of negative mental health outcomes (Cha & Nock, 2014; Klonsky, 2009). Earlier onset of suicidal thoughts or NSSI may also be associated with more severe outcomes through adolescence and adulthood, including suicide plans and attempts (Ammerman et al., 2018; Cha & Nock, 2014), which further emphasizes the need for early risk detection and intervention.

One factor that may be particularly important to children at risk for SITBs is maternal depression. Children of mothers with a history of major depressive disorder (MDD) are at higher risk of developing various forms of psychopathology (for review see Goodman et al., 2011) as well as SITBs (Hammerton et al., 2015) than children of never depressed mothers. Although the exact mechanisms of risk are not clear, children of mothers with MDD are exposed to more stressors inside and outside the home, which could increase risk (Adrian & Hammen, 1993; Feurer et al., 2016). Despite this increased risk, not all children of mothers with depression develop these negative outcomes, highlighting the importance of identifying moderators of risk. Much of the research on moderators has focused on identifying additional risk factors, and relatively little is known about specific protective factors in these higher-risk youth. This type of investigation is important because protective factors may be unique influences rather than just reflecting the absence or low levels of a given risk factor.

Theories of SITB risk in youth highlight the role of peer influences (Kirshenbaum et al., 2024). Thus far, research has focused on negative peer influences such as peer victimization (Tsypes & Gibb, 2016; Victor et al., 2019) or exposure to a peer who engages in SITBs (Hasking et al., 2013; Prinstein et al., 2010; Schwartz-Mette & Lawrence, 2019) and, as noted above, much less is known about potential protective peer influences, especially in the context of children at risk due to a family history of MDD. This said, there is preliminary evidence that peer social support may buffer the impact of stress on risk for SITBs in adolescents (Bertera, 2007; Mackin et al., 2017). However, it is unclear whether specific aspects of the peer environment (i.e., close friendships versus general peer environment) play critical roles in increasing/decreasing risk for SITBs. Due to the breadth of experiences within the peer environment during youth, it is important to examine the role of these different peer relationships, such as support within close friendships versus broader peer groups. Given the salience of peer relationships in the transition from childhood to adolescence, positive relations may protect youth from thinking about or engaging in self-injurious behaviors in this age range, including suicidal ideation, attempts, and NSSI.

Because of the increased risk of SITBs that occurs during the transition to adolescence (Hua et al., 2023; Esposito et al., 2023), our study focused on the impact of different types of peer social support (i.e., close friend versus classmate support) on children and early adolescents at risk for developing SITBs. We tested the following hypotheses in a two-year multi-wave study of children and early adolescents (8–14 year olds): (i) children of mothers with a history of MDD during their lives, compared to children of never depressed mothers, would be at elevated risk for SITBs during the follow-up; (ii) children’s levels of perceived classmate and close friend social support would act as a protective factor, reducing risk for SITBs in both groups of children of depressed mothers and children of never depressed mothers; and (iii) peer support would moderate the effect of maternal MDD, such that higher classmate and close friend support would serve to buffer at least some of the increased SITB risk associated with a maternal history of MDD. We also predicted that each of these effects would be at least partially independent of children’s baseline levels of depressive symptoms. Finally, we conducted exploratory analyses to determine whether any of the effects were moderated by children’s age. From a developmental standpoint, it is possible that maternal MDD and close friend or classmate support function differently across the developmental window of late childhood into early and middle adolescence. During childhood, peer relationships are still developing and the impact of maternal MDD may be more pronounced. However, in early adolescence, when peer interactions become more complex and emotionally significant, the effect of peer support may take on greater importance in buffering risk for SITBs. Therefore, it is crucial to also explore age as a potential moderator in understanding the role of maternal MDD and peer support in the development of SITBs across this broad developmental window.

## Method

### Participants

Participants were 215 mother-child dyads recruited from the community for a study examining risk in offspring of mothers with a history of MDD. To qualify for the study, mothers were required to meet criteria for MDD during their child’s lifetime (*n* = 108) according to the *Diagnostic and Statistical Manual of Mental Disorders – Fourth Edition* (*DSM-IV*; American Psychiatric Association, 2000) or have no lifetime diagnosis of any DSM-IV mood disorder and no current Axis I diagnosis (*n* = 107), and recruitment was designed to ensure approximately equal numbers in each group. Exclusion criteria for both groups of mothers included symptoms of schizophrenia, alcohol or substance dependence within the last 6 months, or a history of bipolar disorder. In addition, children were required to be the biological child of the participating mother and be between the ages of 8 and 14 at the baseline assessment. Only one child per family could participate and, if more than one child per family in this age range was available for participation, one child was chosen at random. The average age of mothers at the baseline assessment was 40.59 years (*SD* = 6.87, Range = 24–55). Of the mothers, 3.72% were Hispanic and, in terms of race, 87.91% were non-Hispanic White, 3.26% were Black, 3.26% were multi-racial, 1.40% were Asian, and the rest were from other racial groups. The average age of children at the baseline assessment was 10.94 years (*SD* = 1.87) and 52.56% were girls. Of the children, 3.72% were Hispanic and, in terms of race, 82.79% were non-Hispanic White, 7.91% were multi-racial, 3.72% were Black, 1.40% were Asian, and the rest were from other racial groups. The median family income was $50,001 to 55,000.

### Procedure

Participants were recruited from the community through a variety of means (e.g. television, newspaper and bus ads, flyers). These ads were developed specifically to recruit mothers with a history of MDD during their child’s life or no lifetime history of any depressive disorder. Interested mothers were screened over the phone to determine potential eligibility, and those meeting inclusion criteria were invited to complete the study. At the baseline assessment, after providing informed consent/assent, participants completed diagnostic interviews and questionnaires (described below). Following this baseline assessment, participants completed follow-up appointments 6, 12, 18, and 24 months after the initial assessment. At each of these assessments, trained interviewers assessed for the presence of SITBs in the children during the previous 6 months. Families were compensated monetarily for their participation. All study procedures were approved by Binghamton University’s Institutional Review Board.

### Measures

The Structured Clinical Interview for DSM-IV Axis I Disorders (SCID-I; First et al., 1995) was administered by trained interviewers at the baseline assessment to determine current and lifetime histories of Axis I disorders in mothers. The Schedule for Affective Disorders and Schizophrenia for School-Age Children – Present and Lifetime Version (K-SADS-PL; Kaufman et al., 1997) was used to assess current and lifetime histories of DSM-IV Axis I disorders in the children. The K-SADS-PL was administered to mothers and their children separately by the same interviewer, while a second interviewer administered the SCID-I (i.e., one interviewer administered SCID-I and the other interviewer administered K-SADS-PL). As noted above, 108 mothers met criteria for at least one episode of MDD during their child’s life. Interrater reliability was assessed by coding a subset of 20 SCID-I interviews by a second interviewer and inter-rater reliability for the diagnoses of MDD was excellent (κ = 1.00).

During the K-SADS-PL interviews at the baseline and follow-up assessments, interviewers assessed for the presence of SITBs in children. More specifically, suicidal ideation was assessed by asking, “Some children who get upset or bad think about dying or even killing themselves. Have you [Has your child] ever had such thoughts?” For any report of suicidal ideation, the interviewer asked follow-up questions, as prompted in the K-SADS interview, to ensure the child specifically experienced passive or active suicidal ideation rather than thoughts of death more generally. Self-injury was assessed by asking, “Did you [your child] ever try to hurt yourself [themselves]?”, and “Have you [your child] actually tried to kill yourself [themselves]?” Follow-up questions were used to differentiate intentional self-injury with (suicide attempt) versus without (NSSI) intent to die. For analyses, we included any reports of child SITBs by the parent or child. The initial assessment focused on children’s lifetime history of SITBs, and each follow-up assessment focused on any behaviors that had occurred since the previous interview. If a family missed a given follow-up assessment, the interviewer assessed any SITBs that may have occurred since the prior visit. Any SITB endorsed was probed for the date of its occurrence, and analyses focused on time until first onset of any SITB during the follow-up. At the baseline assessment, 32 children reported a lifetime history of SITBs (20 children of mothers with MDD). During the two-year follow-up, 67 children reported at least one SITB (44 children of mothers with MDD), for 45 of whom this was the first onset of any SITB (27 children of mothers with MDD).

Children’s levels of social support were assessed using the Social Support Scale for Children (Harter, 1985), which was administered at the baseline assessment. The SSSC was developed for use in children and adolescents aged 8–18 (Harter, 1985). It includes two six-item subscales of peer support: classmate support and close friend support. Each of the subscales indicates the levels to which the child feels cared for. The classmate support subscale identifies how caring, friendly, and inclusive classmates are, and the close friend subscale identifies if the child has a close friend who listens, understands, and spends time with them. For each item, children were asked to decide which of two opposing sentences was most like them, and then rate this item as “Really True for me” or “Sort of True for me” (e.g., “Some kids have a close friend who they can tell problems to” BUT “Other kids don’t have a close friend who they can tell problems to”). Each item was scored on a 4-point scale, with responses averaged across items and higher scores reflecting higher levels of social support. Previous studies have supported the reliability and validity of the SSSC in youth (Harter, 1985; Lipski et al., 2014). In the current sample, the internal consistency was α = 0.78 for classmate support and α = 0.80 for close friend support. A subset of children (*n* = 191) completed the SSSC six months after the baseline assessment (*M* = 6.20 months, *SD* = 0.87) and the retest stabilities of classmate support and close friend support were *r* = 0.63 and 0.42, respectively (both *p*s < 0.001).

Finally, children’s symptoms of depression were assessed at the baseline assessment with the Children’s Depression Inventory (CDI; Kovacs, 1981) for use in sensitivity analyses. The CDI is a self-report measure that has demonstrated strong psychometric properties in community samples of children and adolescents (Smucker et al., 1986). In the current study, the CDI demonstrated good internal consistency (α = 0.86).

### Analysis Plan

We used Cox regression survival analyses to examine the unique and interactive effects of mothers’ MDD histories and children’s levels of social support from peers in predicting their risk for SITBs during the 2-year follow-up. Specifically, we examined time until the first incident of any form of SITB during the follow-up. Using a hierarchical approach, we entered mother MDD group in the first block of the survival analysis and then added one of the two forms of social support (support from close friends or support from classmates) in the second block separately. This allowed us to determine whether children of mothers with a history of MDD during their lives were at increased risk for SITBs during the follow-up (block 1) and whether children’s levels of either close friend or classmate peer support predicted unique variance in risk beyond that accounted for by mothers’ MDD history. Then, in block 3, we added the mother MDD × social support interaction, which allowed us to determine whether either type of peer social support moderated the link between mothers’ MDD history and children’s risk for SITBs. We accounted for right-censoring in our survival analysis models, such that participants who did not report SITBs by the end of the study period or were lost to follow-up were treated as censored at their last available time point.

Follow-up sensitivity analyses were conducted to determine whether any significant relations were at least partially independent of potential confounding variables including children’s levels of depressive symptoms at the baseline assessment and children’s history of SITBs prior to the baseline assessment. Given the relatively wide age range of the sample, we also conducted exploratory analyses to determine whether any of the relations examined were moderated by child age.

## Results

Of the 215 families who completed the baseline session, 200, 189, 164, and 165 completed the 6-, 12-, 18-, and 24-month follow-ups, respectively, and 89.30% completed at least 3 of the 5 assessments. We examined whether participants who missed one or more assessments differed from those with complete data at all assessments on any demographic or clinical variables and none of these analyses were significant (lowest *p* = .17). As noted above, participants who did not complete the full follow-up period were considered right censored in the survival analyses. Preliminary analyses revealed that the questionnaire variables exhibited significant negative (both social support variables) or positive (depressive symptoms) skew. These variables were transformed to satisfy assumptions of normality prior to further analysis (inverse: SSSC-classmate and SSSC-close friend; square root: CDI). Correlations and descriptive statistics for study variables are presented in Table 1. To facilitate comparison with other studies, the means and standard deviations presented are based on untransformed variables.

**Table 1:** Correlations among study variables

	1	2	3	4	5	M (SD) or %
1. Child age	-					10.94 (1.87)
2. Child sex	0.03	-				52.56%
3. Mother MDD	0.04	− 0.11	-			50.23%
4. SSSC-CS	− 0.06	0.08	− 0.20**	-		3.31 (0.60)
5. SSSC-CF	0.01	0.18**	− 0.16*	0.56**	-	3.58 (0.52)
6. SITB	− 0.05	0.02	0.21**	− 0.29**	− 0.11	31.16%

Child sex is coded as 1 = girl and 0 = boy. *MDD* Major depressive disorder (1 = yes, 0 = no). *SSSC-CS* Social Support Scale-Classmate Support. *SSSC-CF* Social Support Scale-Close Friend. *SITB* Self-injurious thoughts or behaviors during the follow-up (1 = yes, 0 = no)

*p <.05. ** *p* <.01

**Fig. 1:**
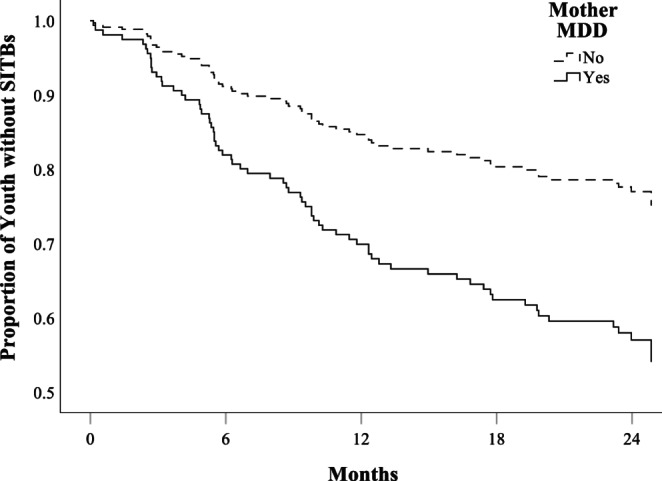
Results of survival analyses predicting time to onset of self-injurious thoughts and behaviors (SITBs) as a function of mothers’ history of major depressive disorder (MDD)

Next, we conducted the survival analyses to examine risk for SITBs during the follow-up. In the first block of the survival analysis, we found a significant main effect of mother MDD, *Wald* = 8.14, *p* =.003, *Hazard Ratio (HR)* = 2.05 (95% confidence interval [CI] = 1.07, 3.01), indicating that children of mothers with a history of MDD were over twice as likely to develop SITBs during the follow-up than children of never depressed mothers (see Fig. 1). As noted above, levels of social support from close friends and from classmates were added to the second block of separate analyses. Levels of support from classmates, *Wald* = 16.68, *p* < .001, *HR* = 0.49 (95% CI: 0.34, 0.69), but not close friends, *Wald* = 0.82, *p* = .37, *HR* = 0.82 (95% CI: 0.53, 1.26), accounted for significant unique variance in children’s risk for SITBs beyond that explained by mothers’ MDD history. Finally, in the third block, neither the mother MDD × classmate support, *Wald* = 0.15, *p* = .70, *HR* = 1.17 (95% CI: 0.51, 2.69), nor the mother MDD × close friend support, *Wald* = 0.44, *p* = .51, *HR* = 0.70 (95% CI: 0.24, 2.01), interactions were significant indicating that neither form of social support significantly moderated the link between maternal MDD and children’s risk for SITBs. Thus, mothers’ MDD history and children’s levels of social support from classmates were individual rather than interactive predictors of risk.

We then conducted sensitivity analyses to determine whether the observed relations were at least partially independent of children’s levels of depressive symptoms at the baseline assessment by adding CDI scores as a covariate to the survival analyses. In this model, CDI score significantly predicted children’s risk for SITBs during the follow-up, *Wald* = 8.55, *p* = .003, *HR* = 1.06 (95% CI = 1.02, 1.10). Importantly, even after accounting for the role of baseline depressive symptoms, maternal history of MDD, *Wald* = 4.20, *p* = .04, *HR* = 1.79 (95% CI = 1.03, 3.11), and social support from classmates, *Wald* = 4.03, *p* = .04, *HR* = 0.21 (95% CI = 0.05, 0.96), remained significant.

Given that some of the children had a history of SITBs prior to the baseline assessment, we then examined whether mothers’ MDD history and social support from classmates remained significant predictors of SITBs during the follow-up even after statistically controlling for children’s prior history of SITBs at the baseline assessment. Maternal history of MDD, *Wald* = 5.02, *p* = .03, *HR* = 1.80 (95% CI = 1.08, 3.02), and social support from classmates, *Wald* = 5.45, *p* = .02, *HR* = 0.21 (95% CI = 0.06, 0.78), remained significant predictors even with prior SITB history entered as a covariate in the survival analysis. Although we also examined whether prior history of SITBs moderated the relation between either predictor and risk for SITBs during the follow-up, neither of these interactions was significant (lowest *p* = .11), suggesting that the predictive validity of maternal MDD and social support from classmates is similar for both first onsets and recurrence of SITBs in children.

Next, because our study included a fairly broad age range of youth and the role of social support from peers may differ across these ages, we conducted exploratory analyses to determine whether youth age moderated any of the relations examined.^1^ Child age did not significantly moderate the effect of mother MDD, *Wald* = 2.68, *p* = .10, *HR* = 1.27 (95% CI = 0.95, 1.70), or close friend support, *Wald* = 1.35, *p* = .25, *HR* = 0.71 (95% CI = 0.40, 1.27), though there was a marginal child age × classmate support interaction, *Wald* = 3.83, *p* = .051, *HR* = 0.50 (95% CI = 0.24, 1.002). Although not meeting the formal threshold for statistical significance, we conducted follow-up analyses for interested readers by examining the main effect of classmate support in younger versus older youth (operationalized by a median split based on children’s age at the baseline assessment). In these analyses, classmate support was a stronger predictor of SITB risk in older, *Wald* = 9.43, *p* = .002, *HR* = 0.03 (95% CI = 0.004, 0.30), compared to younger, *Wald* = 4.64, *p* = .03, *HR* = 0.16 (95% CI = 0.29, 0.85), youth.

Finally, although primary analyses focused on SITBs broadly, exploratory analyses examined SI and NSSI separately. During the follow-up, 61 children experienced SI and 16 children had NSSI (thankfully only one child made a suicide attempt during the follow up). In these analyses, mother MDD, *Wald* = 9.67, *p* = .002, *HR* = 2.34 (95% CI = 1.37, 3.99), and social support from classmates, *Wald* = 11.12, *p* < .001, *HR* = 0.10 (95% CI = 0.03, 0.39), but not social support from close friends, *Wald* = 0.34, *p* = .56, *HR* = 0.72 (95% CI = 0.23, 2.20), significantly predicted risk for SI during the follow-up. In contrast, social support from classmates, *Wald* = 9.13, *p* = .003, *HR* = 0.01 (95% CI = 0.0002, 0.16), and close friends, *Wald* = 4.05, *p* = .04, *HR* = 0.10 (95% CI = 0.01, 0.94), but not mother MDD, *Wald* = 1.03, *p* = .31, *HR* = 1.69 (95% CI = 0.61, 4.65), predicted risk for NSSI during the follow up. Statistically controlling for the influence of baseline depressive symptoms, mother MDD, *Wald* = 5.99, *p* = .01, *HR* = 1.96 (95% CI = 1.14, 3.37), continued to significantly predict risk for SI during the follow up, but the effect of social support from classmates was reduced to a nonsignificant trend, *Wald* = 2.84, *p* = .09, *HR* = 0.28 (95% CI = 0.06, 1.23). In predicting risk for NSSI, after statistically controlling for baseline depressive symptoms, neither social support from classmates *Wald* = 3.02, *p* = .08, *HR* = 0.08 (95% CI = 0.001, 1.51), nor close friends, *Wald* = 0.59, *p* = .44, *HR* = 0.39 (95% CI = 0.04, 4.24), remained significant.

## Discussion

This study examined links between maternal history of MDD and risk for the development of SITBs in children, and whether peer social support may help to buffer this risk. As hypothesized, children of mothers with a history of MDD had increased risk for onset of SITBs during the follow-up compared to children of never-depressed mothers. Partially supporting our second hypothesis, social support from classmates acted as a unique, rather than moderating, protective factor predicting reduced risk for SITBs in children even after accounting for the influence of maternal MDD history. Importantly both maternal MDD history and social support from classmates predicted risk for SITBs during the follow-up even after taking into account children’s levels of depressive symptoms at the baseline assessment and prior history of SITBs.

Our findings add to research (e.g., Lee et al., 2020) showing that offspring of mothers with depression are at increased risk for SITBs by demonstrating that this risk is evident by late childhood/early adolescence. The current findings also extend prior research emphasizing the role of peer influences in SITB development (Victor et al., 2019; Schwartz-Mette & Lawrence, 2019) by focusing on specific domains of peer support as well as the influence of peer relationships in reducing rather than increasing the risk of SITBs. We found that higher levels of support from classmates predicted significantly lower risk of SITBs across a two-year follow-up, even after accounting for the influence of mothers’ MDD history. It is notable that classmate support, but not close friend support, was a significant protective factor, suggesting that the broader peer environment may be more salient in reducing risk for SITBs during late childhood and early adolescence. These results also highlight the importance of assessing different sources of support, which could have differential effects across outcomes. Support from classmates may provide a unique sense of belonging and social integration, which may counteract feelings of isolation or rejection that can exacerbate SITBs (Prinstein et al., 2010; Schwartz-Mette & Lawrence, 2019). Additionally, peer group dynamics in school settings might offer children more frequent and diverse social interactions, which could facilitate resilience against SITBs. This finding is consistent with Harter’s (1985) notion that perceptions of classmate support reflect a child’s broader acceptance within their social environment, which could have a stronger protective effect than the more intimate but potentially less consistent support from close friends. This novel finding suggests that broader social inclusion, as reflected by classmate support, may be a critical protective factor for vulnerable youth, potentially because it reflects a more generalized sense of belonging and acceptance within a larger peer group beyond just intimate friendships.

It is important to note that we utilized subjective reports of peer support in our study. Therefore, our conclusions are limited to children’s perceptions of support rather than classmates’ actual behavior. However, perceptions of support also offer a window for intervention, which can be targeted to highlight any aspects of support that might not be recognized by the child and to reduce any cognitive biases (e.g., catastrophizing, overgeneralizing, black-and-white thinking) that may exacerbate the impact of low perceived support. Our results are in line with previous research which has shown that perceived social support from the general peer group is more strongly associated with social-emotional outcomes than perceived support from close friends (e.g., Demaray et al., 2005; Rueger et al., 2016). We should note, however, that we may have had limited power to examine the role of close friend support due to ceiling effects on this variable (40% of children endorsed the maximum value on this variable). This said, our findings add to this growing body of literature highlighting the importance of the classmate environment broadly on positive outcomes throughout adolescence.

Although social support from classmates reduced the risk for SITBs, social support did not significantly moderate or buffer the link between maternal MDD and offspring SITBs. Rather, maternal MDD and social support from classmates appear to be independent predictors of youth risk for SITBs. Previous studies have documented similar trends, where protective influences such as family cohesion or peer connectedness reduce SITB risk in both high- and low-risk populations (Macrynikola et al., 2018).

This study had several strengths, including its relatively large sample size, the multi-wave prospective design, and the use of semi-structured interviews to assess maternal history of MDD and children’s SITBs. Further, to our knowledge, this is the first study to examine the role of specific sources of peer support (i.e. classmate versus close friend) in the development of SITBs in both high-risk and low-risk youth. Despite these strengths, there were also limitations, which highlight important areas of future research. First, we did not examine how changes in peer support over time (e.g., the loss of a close friend) impact SITB trajectories, which would be valuable in elucidating the dynamic nature of social support as a protective factor. Second, although the SSSC was designed validated for use in children and adolescents aged 8–18 years old (Harter, 1985; Lipsky et al., 2014), no studies have formally evaluated measurement invariance of the scale in this age range, so it is possible that the underlying constructs assessed by the SSSC (i.e., perceptions of support from classmates and close friends) differ for youth across different ages. Third, the number of children who reported an onset of SITBs during the study was (thankfully) relatively small, particularly among children of never depressed mothers. This was particularly true for the exploratory analyses examining NSSI and SI separately, which suggested that close friend support may be a stronger buffer for experiences of NSSI than SI. Future research with larger or higher-risk samples are needed to more fully examine these influences, including potential moderating effects. These larger studies should also continue to examine potential age differences in the buffering effects of peer support given our preliminary finding that classmate support may exert a stronger buffering effect in older, compared to younger, youth.

Additionally, although the use of a structured clinical interview, such as the K-SADS, offers significant strengths in assessing psychiatric symptoms, there are potential limitations when it comes to capturing SITBs in youth. Retrospective reporting can be unreliable, especially when assessing events that are emotionally charged or developmentally complex (Klimes-Dougan et al., 2022). We sought to minimize this during the follow-up by only inquiring about the previous six months. In addition, the phrasing of suicide-related questions may significantly impact endorsement rates (Ammerman et al., 2021), and discrepancies between parent and child reports—particularly in younger samples—may further complicate accurate identification of SITBs (DeVille et al., 2020). As such, it is possible that some SITBs went unreported or underreported, particularly among children at the lower end of the age spectrum. These limitations highlight the need for continued development of age-appropriate, multi-informant, and fine-grained tools for suicide risk assessment in pediatric populations (Cwik et al., 2020).

In summary, the current study contributes to the understanding of risk for the emergence of SITBs in children. The findings highlight the importance of considering maternal MDD history in offspring risk of SITBs, as well as considering the buffering effects that a positive peer environment has on children’s risk. Our findings may have some important clinical implications for early SITB prevention in youth. First, the robust association between maternal MDD and youth SITBs highlights the importance of assessing family mental health histories as part of early screening for suicide risk. Interventions aimed at supporting maternal mental health may have downstream benefits for child well-being, particularly in preventing the emergence of SITBs. Second, because peer support from classmates was an independent predictor of risk, regardless of mothers’ MDD history, understanding the broader social context remains essential. This suggests that clinicians should assess the quality of peer relationships more broadly in treatment planning rather than just focusing on close friendships, especially during early adolescence. For example, our finding that social support from classmates helps to mitigate risk for SITBs in youth suggests that interventions focused on promoting positive relationships at school may be effective in decreasing future SITB risk. School-based programs designed to enhance social inclusion and promote peer empathy could be particularly beneficial in preventing SITBs (Berger et al., 2019; Dobias et al., 2023). When youth feel supported in school, they may be more likely to engage in positive coping strategies to deal with negative emotions or feel more comfortable seeking support from trusted individuals in their lives.

## Data Availability

Data reported in this article are avail­able upon request from the corresponding author

## Footnotes

1 We thank an anonymous review for suggesting these analyses.
